# Imaging of non‐accidental injury; what is clinical best practice?

**DOI:** 10.1002/jmrs.269

**Published:** 2018-03-24

**Authors:** Amy Nguyen, Robin Hart

**Affiliations:** ^1^ Department of Medical Imaging and Radiation Sciences Faculty of Medicine Nursing and Health Sciences Monash University Clayton Victoria Australia

**Keywords:** Child abuse, imaging, non‐accidental injury, radiography

## Abstract

Non‐accidental injury (NAI) remains the leading cause of morbidity and mortality in children. Fractures are the second most common findings of NAI, after cutaneous lesions such as bruises and contusions. Imaging in NAI remains a controversial issue with little agreement concerning how, when and what imaging modalities should be used in the investigation of suspected cases. This review addresses the radiological investigations and findings of NAI, and the differential diagnoses of these findings. Adherence to the international guidelines for skeletal survey imaging is recommended. This ensures the content and quality of the radiographic series are of an optimal standard to improve the detection of occult fractures, and ensuring the accurate reporting of images. The involvement of a paediatric radiologist is important, if not essential in the diagnosis of NAI. In the evaluation of suspected cases, the role of the radiologist includes the detection of radiological findings suggestive of NAI, and the differentiation of these findings from normal variants and underlying pathologies. The diagnosis of NAI relies not only on radiological imaging, but also a combination of clinical and social findings. It is mandatory that all physicians work in close collaboration to improve diagnostic accuracy, as failure to diagnose NAI carries significant risk for morbidity.

## Introduction

Non‐accidental injury (NAI) can be defined as an abusive act by a caregiver leading to injury of a child.^1^ It remains the leading cause of morbidity and mortality in children.^2^, ^3^ In Australia, 42,457 children were abused or neglected, of which 18% were physically abused.^4^ Young children are at greater risk of NAI, and primary caregivers are often the perpetrators of abuse.^5^

The investigation of NAI requires a thorough history and clinical examination, which may be supplemented by radiological investigations, including radiographic and cross‐sectional imaging.^6^ Imaging in NAI remains a contentious issue, with little concordance regarding how, when and what imaging modalities should be used in the workup of the child who is suspected of having suffered abuse.^7^ Failure to diagnose NAI carries significant risk for morbidity, particularly in non‐ambulatory and nonverbal children.^3^

## Aim

This review identifies clinical best practice for the imaging of suspected NAI from the medical and legal perspective. Additionally, the role of the radiographer and the social issues associated with non‐accidental injury examinations are discussed.

## Methods

A literature search of the databases CINAHL Plus, Ovid MEDLINE, PubMed and Scopus were performed to obtain publications discussing the imaging of NAI. The key search terms consisted of:

- non‐accidental injury (NAI)
- non‐accidental trauma (NAT)
- battered child syndrome (BCS)
- shaken baby syndrome (SBS)
- child abuse
- paediatrics OR pediatrics
- radiography
- radiology and
- imaging.

The search was limited to articles published within the last 6 years. Articles not in English were excluded.

The initial search found 94 articles that met the inclusion criteria, which were selected for abstract review. Articles were further excluded after a closer review did not report quantitative findings specific to NAI, and did not add additional information to the articles already selected. From the abstracts, 20 primary articles were selected. Additional articles were identified by reviewing the reference lists of the primary articles.

## Results

A thematic analysis of the results yielded the following areas for consideration:

### Imaging guidelines

A study using a web‐based survey by Hulson et al.^7^ looked at responses from 134 institutions and found significant variations in the guidelines for radiographic and cross‐sectional imaging in the investigation of suspected NAI. There are two current guidelines: the American College of Radiology (ACR) and the Society for Pediatric Radiology (SPR) ‘Practice guideline for skeletal surveys in children’ and the Royal College of Radiologists (RCR) and the Royal College of Paediatrics and Child Health (RCPCH) ‘Standards for radiological investigation of suspected non‐accidental injury’.

Since the publication of the RCR‐RCPCH guidelines, a study by Patel et al.,^6^ looking at 100 skeletal surveys, found that 51% of studies contained all recommended views and each view had an average quality score 97%. A retrospective evaluation by Weldon and Price,^8^ which analysed 121 NAI skeletal survey examinations, found the implementation of imaging checklists improved skeletal survey quality.

The ACR ‘Appropriateness criteria’ provides imaging pathways for the investigation of suspected NAI. Wood et al.^9^ which reviewed 240 cases of skeletal surveys for appropriateness, found that the skeletal surveys were ‘appropriate’ for 80% of cases and ‘necessary’ for 92% of cases.

### Imaging and dose

A retrospective descriptive study by Powell‐Doherty et al.,^3^ looking at 110 skeletal surveys of suspected NAI, identified 79% of studies revealed positive findings in the initial skeletal survey, and no new follow‐up findings. The authors found that initial skeletal surveys are nearly six times more likely to identify positive findings. A retrospective cross‐sectional study by Jha et al.,^5^ which looked at the skeletal surveys of 530 children, identified no fractures on pelvic radiographs and less than 0.2% fractures on lateral spinal radiographs.

A retrospective meta‐analysis by Shelmerdine et al.,^10^ which examined 288 fractures in 281 children, identified non‐supracondylar humeral fractures in 3% and femoral fractures in 9%, of which 11% and 24% were respectively referred for a skeletal survey.

The yield of follow‐up skeletal surveys is variable and complicated by differences in follow‐up imaging. A prospectively planned secondary analysis by Harper et al.,^11^ looking at 2,049 skeletal surveys for suspected NAI, found 51% were recommended for, and of which only 39% had follow‐up skeletal surveys. The authors noted broad variability in the recommendation and completion of follow‐up skeletal surveys in suspected NAI cases. A retrospective descriptive study by Singh et al.,^12^ which looked at 169 follow‐up skeletal surveys, identified new findings in 14% of cases. A prospectively planned secondary analysis by Harper et al.,^13^ looking at 796 follow‐up skeletal surveys due to suspected NAI, found new findings in 21.5% of cases and reassuring findings in 6.9% of cases.

With the evidence of potential radiation risks, some authors have considered the performance of a limited follow‐up skeletal survey. A study by Hansen et al.,^14^ looking at 534 cases of suspected NAI, found that a limited follow‐up skeletal survey, omitting the spine and pelvic views, missed 0.2% of new findings. The authors noted no clinically significant difference between the complete and the limited view follow‐up skeletal surveys. Berger et al.^2^ using a Monte Carlo simulation, estimated the total effective dose of a skeletal survey for a child (aged less than 12 months) to be 0.2 mSv.

Considering the failure of attendance for follow‐up imaging, some authors have suggested the use of other imaging modalities to complement the radiographic skeletal survey. A retrospective analysis by Bainbridge et al.,^15^ looking at 166 studies including both skeletal survey and bone scintigraphy, found bone scintigraphy added confidence in 8% of studies and identified new findings in 12% of studies.

A retrospective cross‐sectional study by Culotta et al.,^16^ examined 177 studies of suspected abusive head trauma and showed that 35% of studies had skull fractures identified on skull radiographs and 38% by CT. The authors found no significant difference between the sensitivity of skull radiographs and head CT with 3D reconstructions in identifying skull fractures. Sanchez et al.^17^ reviewed the examinations of four children requiring CT of the chest in addition to the skeletal survey, and found that the average effective dose for reduced‐dose chest CT was 0.56 mSv.

### Assessment of reporting

A delayed diagnosis or misdiagnosis of NAI can have devastating consequences. The study by Karmazyn et al.,^18^ which looked at 178 skeletal surveys for suspected NAI, demonstrated that a double‐read found a 4.5% discrepancy in the skeletal survey findings. The authors found that limiting double‐read to initially positive studies improves diagnostic accuracy. A retrospective review by Jackson et al.,^19^ looking at 18 cases of delayed diagnosis of NAI, found several contributing factors categorised as clinical or system‐based limitations.

## Discussion

### Education

All physicians should be able to recognise NAI and respond accordingly.^19^ However, inconsistencies and under‐reporting of NAI still widely exists.^10^ Education is the best approach to aid management of future suspected NAI cases.^10^ Educational interventions for emergency department physicians, nurses and radiologists to improve recognition of, and response to, NAI, and improve outcomes for children.^19^

With variability in medical education of NAI, contributing clinical factors, such as inadequate assessment of signs and symptoms and misdiagnoses from radiological investigations, can be used as learning outcomes for continuing medical training.^19^ The implementation of regular NAI training sessions for emergency department staff have ensured all specialties remain up‐to‐date with current NAI practices.^10^ Increased education and optimised clinical and radiological protocols can improve awareness of NAI.^10^

### Recognition

There is no ‘typical’ presentation of an abused child.^20^ Fractures through the entire skeleton are the second most common findings of NAI, after cutaneous lesions such as bruises.^18^, ^21^, ^22^, ^23^, ^24^ Abusive fractures are more common in children under 18 months old.^23^ Suspicion of NAI increases when the mechanism of injuries are discordant with the caregiver's history and the child's developmental status, or when occult fractures are discovered.^22^

### Investigation

There is no ‘gold standard’ in the diagnosis of NAI,^20^ however, the radiological detection of injuries is integral in the diagnosis of NAI.^15^

#### Conventional radiography

Conventional radiography continues to be the mainstay in the investigation of suspected NAI, both in identifying and in the work‐up of suspected NAI cases.^1^, ^21^ A skeletal survey is a systematically performed series of high quality radiographs demonstrating the entire skeleton, and is routine in the assessment of children under 2 years old.^3^, ^8^, ^21^, ^24^, ^25^ Skeletal surveys are performed to identify occult fractures, exclude underlying skeletal dysplasia or metabolic conditions, and aid in fracture dating.^1^ The skeletal survey must be performed at an optimal standard of technical quality using high detail imaging systems, with radiographs acquired following a rigorous protocol, with special consideration to patient positioning, centring and collimation.^25^, ^26^, ^27^ This ensures that radiographs have the required detail to detect subtle fractures whilst keeping the patient radiation dose ‘as low as reasonably achievable’.^25^ A ‘babygram’, whole body radiograph, must never be performed.^1^, ^25^

International guidelines for skeletal survey have been published by the American College of Radiology and the Society for Pediatric Radiology (ACR‐SPR) and the Royal College of Radiologists and the Royal College of Paediatrics and Child Health (RCR‐RCPCH) (Table 1).^21^ According to both guidelines, a complete radiographic series comprises of at least 20 images.^21^ Adherence to the full skeletal survey is recommended.^22^ The radiographic series including oblique views of the ribs remains the gold standard in the detection of occult fractures in NAI.^25^

**Table 1:** Imaging guidelines for skeletal survey in suspected NAI

ACR‐SPR	RCR‐RCPCH
Thorax (AP and lateral), to include ribs,^a^ thoracic and upper lumbar spine Pelvis (AP), to include the mid lumbar spine Lumbosacral spine (lateral) Cervical spine (AP and lateral) Skull (frontal and lateral), additional views if needed – oblique or Towne view Humeri (AP) Forearms (AP) Hands (PA) Femora (AP) Lower legs (AP) Feet (AP or PA)	Thorax (AP), right and left oblique views of the ribs Pelvis (AP) Lumbosacral spine (lateral) Cervical spine (lateral) Skull (frontal and lateral), Towne view if occipital injury suspected Humeri (AP)^b^ Forearms (AP) Hands (PA) Femora (AP) Lower legs (AP) Feet (AP)

NAI, non‐accidental injury; ACR‐SPR, American College of Radiology‐Society for Pediatric Radiology; RCR‐RCPCH, Royal College of Radiologists‐Royal College of paediatrics and Child Health; AP, anteroposterior; PA, posteroanterior.

a Oblique views recommended, but not routine.

b Lateral coned views of the elbows, wrists, knees and ankles may demonstrate metaphyseal injuries in greater detail. The consulting radiologist should decide this at the time of checking the films with radiographers.

In cases of equivocal findings, a follow‐up skeletal survey obtained after 10–21 days, which excludes the skull, has increased sensitivity and specificity for healing fractures.^11^, ^21^ This can increase the likelihood of detecting occult fractures not visible at the time of the initial radiographic series, can explain indeterminate findings, and can be valuable in estimated dating of fractures.^15^, ^25^

The American Academy of Pediatrics (AAP) recommends performing a follow‐up skeletal survey within 10–21 days after the initial skeletal survey if ‘abuse is strongly suspected on clinical grounds’ and ‘when the initial findings are abnormal or equivocal’.^28^ If the possibility of NAI is eliminated, no follow‐up imaging is required.^3^

Follow‐up skeletal surveys can vary from a single chest radiograph to a complete skeletal survey.^24^ Elimination of any radiograph for a limited follow‐up skeletal survey must be balanced with the possibility of missing occult fractures.^24^ As a minimum follow‐up examination, a chest radiograph is performed to identify healing rib fractures.^1^ Views of the skull, pelvis and lateral spine may be performed as indicated by clinical signs and symptoms.^5^

Child abuse is not limited to the abused child and may be directed to all siblings.^21^ When a child presents for suspected NAI, the siblings living in the same conditions are also assessed.^1^, ^26^ Siblings under 2 years old are routinely assessed with a skeletal survey.^1^, ^21^

All examinations should be performed by two radiographers experienced in radiographic paediatric imaging.^1^ The skeletal survey must be supervised by a radiologist, ideally trained in paediatrics, who will also advise on additional views.^1^ Once all images have been reviewed by the consulting radiologist, the examination is considered complete.^26^, ^27^ Lead anatomical markers should be used for added credibility, however the use of electronic markers applied during image post‐processing is also adequate.^23^ Gonadal shielding can obscure fractures of the pubic rami, therefore is not used with the initial skeletal survey.^23^ The radiographers are identified on the images, either by their initials or by pre‐allocated codes.^27^

The content and quality of the skeletal survey should be consistent between institutions, to improve the detection and ensuring accurate reporting of the images.^6^

#### Bone scintigraphy

Bone scintigraphy is used in cases of suspected NAI to complement the skeletal survey, either when safety concerns remain or if failure to attend for follow‐up imaging is likely.^15^, ^21^, ^22^ It has an increased diagnostic yield in anatomically complex locations, such as the ribs, scapulae, spinal column, pelvis and hands and feet.^15^, ^21^ Bone scintigraphy demonstrates pathophysiological abnormalities; positive sites require confirmatory radiographs.^1^, ^23^

In the ACR ‘Appropriateness criteria’, no consensus on the use of bone scintigraphy was reached; it is ‘indicated when a clinical suspicion of abuse remains high and documentation is still necessary’.^29^

#### Computed tomography

Computed tomography (CT) offers the advantages of 3D imaging with volumetric and multi‐planar reconstructions.^21^, ^22^ Given the relatively high radiation doses involved, CT should not be used in place of conventional radiography, and should be restricted to critically ill children who may need neurosurgical intervention.^21^ Iterative reconstruction and all appropriate dose reduction techniques should be used to reduce radiation exposure.^22^

Non‐contrast‐enhanced CT is the imaging modality of choice for suspected head trauma, and has the advantage of being readily accessible with relatively quick acquisition times.^1^, ^23^, ^25^ It is highly sensitive and specific for the detection of acute cranial injury, intracranial haemorrhage, and secondary changes such as cerebral oedema and infarction.^1^, ^23^, ^25^ Fractures and soft tissue swelling can also be diagnosed on CT using appropriate window settings.^26^ Furthermore evaluation with MRI may be helpful in the setting of an abnormal CT examination.^22^

Chest CT is highly sensitive at identifying fractures at all stages of healing, but exposes the child to significantly higher radiation dose than a chest x‐ray.^23^ Contrast‐enhanced CT of the chest and abdomen is the mainstay for imaging of thoraco‐abdominal injuries.^25^ CT of the abdomen and pelvis may be appropriate, particularly if there is suspicion of solid organ or visceral injury.^22^ There are no specific radiologic findings of abusive trauma in the abdomen or pelvis; however, unexplained serological evidence of solid organ insult may prompt imaging.^22^

#### Magnetic resonance imaging

Magnetic resonance imaging (MRI) is highly sensitive to parenchymal injury and allows accurate mapping and detection of subacute and chronic haematomas.^1^, ^25^ For non‐acute head injury presentations, MRI of the brain is the investigation of choice.^24^ In cases of potential abusive head trauma, MRI can be useful in determining whether a haemorrhagic subdural collection contains haemorrhagic products.^22^ Follow‐up MRI is often indicated when an initial CT demonstrates a previously undocumented intracranial injury.^22^

If the initial head CT is abnormal or there is ongoing neurological concern, an additional MRI should be performed as it may provide prognostic information to assist in the management of the child.^24^ Head MRI with diffusion weighted imaging is recommended at 3–5 days and 3–6 months after the initial injury, and enables an in‐depth neuro‐radiological evaluation.^23^, ^24^, ^25^

#### Other imaging modalities

Ultrasound is used as an extension of the physical examination and as an adjunct to traditional imaging methods.^25^ Flourine 18‐labelled sodium fluoride (18F‐NaF) positron emission tomography, with its high spatial resolution, has advantages in bone imaging.^30^ These techniques are performed on a case‐by‐case basis.^21^

#### Radiation dose considerations

There are growing concerns about the small but potential adverse effects of radiation in the paediatric population as a result of imaging investigations of NAI.^24^ All standard radiological investigations for suspected NAI result in radiation exposure for the child.^23^ Children are exposed to radiation at a crucial stage in biological development^3^ and radiation effects have significant time to develop.^5^

Radiation doses in the range 0–100 mSv are considered low, however the ‘linear no‐threshold model’ suggests a risk exists even at low doses.^24^ The effective dose of a skeletal survey using digital radiography is estimated to be 0.2 mSv in infants up to 12 months old.^2^ The effective dose of a skeletal survey in children less than 2 years old is 0.8 mSv.^15^ With an effective dose for bone scintigraphy of 3 mSv in all age groups^15^ and a head CT of 1.9 mSv in a child up to 2.5 years old.^24^ Such low doses suggest that radiation should not be an overriding factor when deciding whether a skeletal survey is needed in suspected NAI cases.^2^ The risk of missed injuries and potentially returning a child to an abusive environment is the primary consideration.^23^

#### The radiographer

Radiographers have a duty to protect and promote the health and welfare of children.^31^ In Australia, mandatory reporting of suspected NAI cases also applies to radiographers.^19^ While it is unlikely that radiographers are to provide a statement in court, the radiographs and subsequent radiological report will often be used as evidence.^31^ The role of the radiographer is to produce optimal quality images with accurate documentation.^25^ During the examination of suspected cases, radiographers must maintain their professionalism and avoid passing judgements.^31^

### Reporting

#### Radiologic findings

Rib fractures in infants and toddlers are generally seen as the hallmark finding of NAI, particularly in cases of abusive head trauma.^21^, ^22^, ^27^ Rib fractures tend to be bilateral and are commonly seen in the posterior or axillary aspect of the rib, but can be found in any location along the rib.^1^, ^22^ Anterior rib fractures is associated with abdominal injury.^1^ The specific mechanism of injury for rib fractures seen in NAI cases is anterior‐posterior compression of the chest,^1^, ^21^, ^22^ where excessive force applied to the ribs over the transverse spinal processes result in posterior rib fractures.^21^ The child is also often vigorously shaken.^1^

Classic metaphyseal lesions (CML) are highly specific finding of NAI.^1^, ^5^, ^21^ CMLs are also called ‘corner’ or ‘bucket handle’ fractures.^21^, ^22^, ^25^ These fractures are commonly seen in the distal femur, proximal and distal tibia/fibula and proximal humerus.^1^, ^21^ The mechanism of injury involves shearing and rotational forces to long bone leading to avulsion fracture of the metaphysis.^22^, ^27^ CMLs generally heal through bone absorption without associated radiographic findings.^1^, ^21^

Abusive head trauma (AHT) is the most common finding of NAI leading to death in children under 1 year old.^22^ Even a low suspicion of AHT should require appropriate follow‐up imaging given the morbidity and mortality of traumatic intracranial injury.^22^ Injuries includes collision with a stationary object, direct impact to the head, and alternating acceleration and deceleration.^22^ Infants are particularly at risk for traumatic brain injury as a result of being shaken due to the relative weight of the head compared to the rest of the body, and relative lack of strength of the neck musculature.^22^ Additional physical examination findings such as retinal haemorrhage may raise suspicion of NAI, but discordance of injury patterns with the caregiver's history is often the leading factor prompting the appropriate investigation.^19^, ^22^

While subdural haematomas are the most common intracranial finding in NAI, its non‐specific nature requires careful correlation with clinical symptoms and patient history.^19,^ 22 Extra‐axial fluid collections are often seen in AHT, and their age is of particular importance.^22^ While the presence of a high attenuation extra‐axial fluid collection is congruent with acute haematoma, a homogenously low attenuation extra‐axial fluid collection may present in the acute setting of arachnoid shearing.^22^ Because of these confounding variables, the use of CT in the dating of extra‐axial fluid collections must be reserved for cases with available previous imaging for comparison.^22^ Physicians should be aware of the presence of abusive spinal injury including its potential coexistence with AHT.^23^ Spinal injury due to NAI can present as fractures, haematoma, soft tissue and neurological injury.^23^

Linear skull fractures are lower in specificity of NAI, and the presence of a cranial fracture is not always associated with intracranial findings.^22^ However, an isolated skull fracture in a child without a substantiated accidental cause should warrant a skeletal survey to exclude additional injury.^22^ AHT may be indicated with the presentation of multiple fractures, bilateral fractures, and fractures crossing suture lines.^22^, ^23^

#### Dating fractures

Healing fractures follow a recognised sequence of histopathological changes, corresponding to radiological features represented on a continuum.^1^, ^23^ The dating of fractures remains an inexact science, and the radiological estimates are in terms of weeks.^23^ However, radiology can distinguish recent from old fractures.^27^ Initially subperiosteal bone formation is seen followed by loss of fracture line definition, soft callus and hard callus formation and finally bone remodelling.^1^

Skull and metaphyseal fractures are more challenging to date, due to lack of callus formation.^1^

#### Differential diagnoses

In cases of suspected NAI, a differential diagnosis must always be considered.^21^ Physicians must consider conditions related to collagen production, bone mineralisation and other pathologies resulting in bone fragility.^21^, ^23^ There are also many conditions which may mimic NAI, such as skeletal dysplasias, osteomyelitis and sickle cell disease.^21^ The rare disorder of congenital indifference to pain, asymbolia, has similar radiological appearances to NAI.^32^ Menkes syndrome, a rare metabolic condition involving copper storage, is identified using genetic studies and hair analysis, and can produce overlapping findings with NAI.^22^

To differentiate NAI from fractures due to underlying pathologies, correlations can be made to prior radiographic studies, serologic testing and physical examination.^22^ Here lies the importance of the skeletal survey, as metabolic disorder generally present in a predictable diffuse pattern, whereas traumatic injury tends to be focal.^22^

#### Radiological reporting

The diagnosis of NAI relies not only on radiographic imaging, but also a combination of clinical, investigative and social findings.^19^, ^21^ Radiologic findings are a significant factor in the decision‐making of physicians about reporting to child protective services.^26^ Missing a fracture in cases of suspected NAI can have devastating consequences.^18^ Double reading in cases of suspected NAI helps ensure injuries are neither missed nor over‐called.^18^, ^33^

In suspected NAI cases, the role of the radiologist is not only in the detection of radiological findings, but also the differentiation of these findings from normal variants and other conditions, determination of fracture ageing, and suggestion to mechanism of injury.^18^ Radiological reporting must state the adequacy of imaging, describe anomalies, rule out any differential diagnoses, and communicate the suspicion of NAI.^26^ The degree of certainty of NAI must also be accurately communicated to physicians and thus to child protective services.^26^

### Medico‐legal implications

Laws governing the health and welfare of children vary between countries.^32^ In Australia, all physicians are legally mandated to report cases of suspected NAI.^19^ If NAI is suspected, child protective services is notified and civil court proceeding begin.^1^ This can take several months before judgement is formed, due to pending reports, court hearings and evaluations.^33^ While the child is moved to a position of safety, child protective services, the police and the court will act in the child's best interest.^1^

Since physicians may be required to provide legal statements in court, it is mandatory that they work in close collaboration to ensure that radiographic images are obtained at the highest possible standard, and accurately reported in an informed manner.^25^ The social and legal outcomes of NAI are associated with the number, extent and severity of the injuries, and requires enhanced recognition.^15^

## Conclusion

Clinical best practice in the radiological imaging of suspected NAI must be performed with rigour to ensure the diagnosis is accurately identified, and has been differentiated from other pathologies that may mimic NAI.^26^ When suspected NAI is encountered, selecting the appropriate radiological investigation is essential to the screening and diagnostic role.^22^ Careful correlation between physical examination findings, the reported history, and the radiologic investigation must be considered in each case.^22^

## Conflict of Interest

The authors declare no conflict of interest.
